# Adverse Clinical Events at the Injection Site Are Exceedingly Rare After Reported Radiopharmaceutical Extravasation in Patients Undergoing ^99m^Tc-MDP Whole-Body Bone Scintigraphy: A 12-Year Experience

**DOI:** 10.2967/jnumed.122.264994

**Published:** 2023-03

**Authors:** Ashwin Singh Parihar, Lisa R. Schmidt, John Crandall, Farrokh Dehdashti, Richard L. Wahl

**Affiliations:** 1 Mallinckrodt Institute of Radiology, Washington University School of Medicine, St. Louis, Missouri; and; 2 Siteman Cancer Center, Washington University School of Medicine, St. Louis, Missouri

**Keywords:** extravasation, infiltration, radiotracer, radionuclide, MDP, bone scan

## Abstract

The deleterious effects of high-dose radiation on normal tissue are sometimes extrapolated to diagnostic (SPECT and PET) radiopharmaceutical extravasation (RPE). It has been hypothesized that diagnostic RPE can have gradually evolving local tissue injury and a potentially increased risk of local dermatologic or oncologic diseases over a longer period. However, data on clinical adverse events after diagnostic RPE are limited. Therefore, our primary aim was to study the occurrence of short-term and long-term clinical adverse events in patients who underwent ^99m^Tc-methylene diphosphonate (^99m^Tc-MDP) whole-body bone scintigraphy (WBBS) with reported RPE. **Methods:** The records of ^99m^Tc-MDP WBBS performed from June 2010 to January 2022 were retrospectively examined for RPE documented in the scan reports. The clinical records of patients with a documented RPE were extensively reviewed for any related short-term adverse events (within 2 wk of the WBBS: local symptoms and care sought for local dermatologic or musculoskeletal issues) and long-term adverse events (until the last follow-up: local deleterious effects and related consults for dermatology, plastic surgery, oncology, or orthopedics). **Results:** Retrospective review of the records of 31,679 ^99m^Tc-MDP WBBS studies showed RPE documented in 118 (0.37%). Medical records were not retrievable for 22 patients, yielding a final cohort of 96 patients with reported RPE. The median follow-up was 18.9 mo (interquartile range, 7.8–45.7 mo). Short-term events were noted in 4 patients, of whom one was asymptomatic. Of the 3 symptomatic patients, 2 experienced mild discomfort at the injection site, and 1 had tender swelling. Three of the 4 events were in patients who had a prior intravenous contrast extravasation for contrast-enhanced CT performed earlier during the day and a ^99m^Tc-MDP injection later at the same site, likely leading to RPE. None of the long-term local events had any plausible link with the RPE event. **Conclusion:** Reported RPE was rare, and 3 patients (0.009%) had short-term local symptoms, all of which were likely related to the prior higher-volume intravenous contrast extravasation. The smaller-volume diagnostic radiopharmaceutical injections for WBBS are highly unlikely to cause local symptoms on their own. No patient had any long-term adverse event with a plausible link to the RPE.

## Visual Abstract

**Figure:**
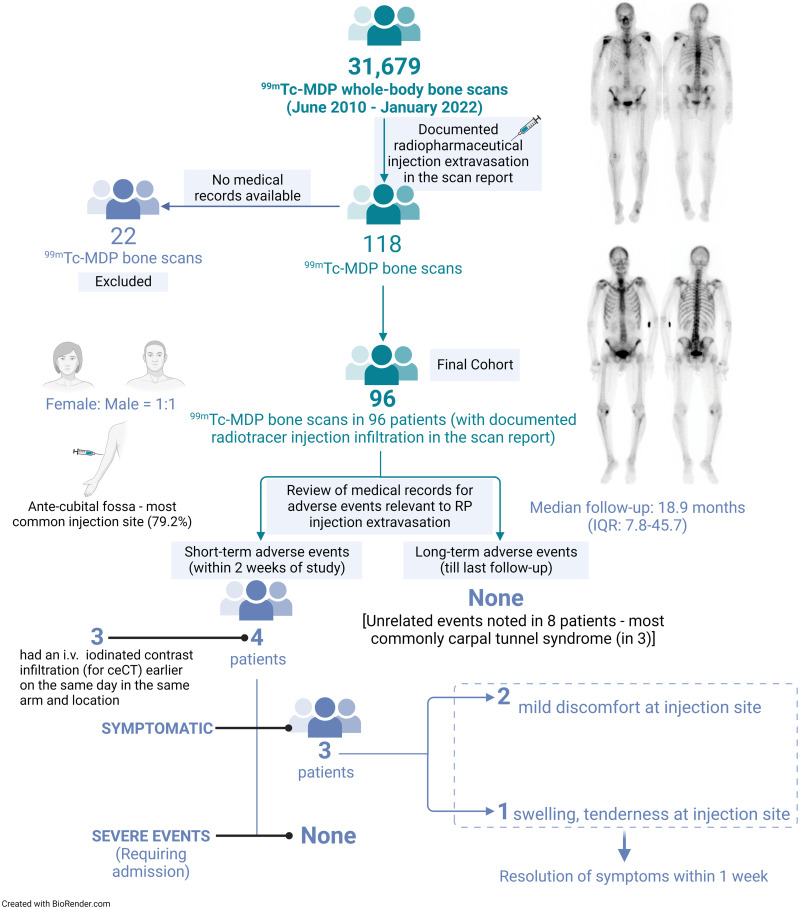

Radiopharmaceutical extravasation (RPE) refers to the unintended leakage of the radiopharmaceutical into the tissue surrounding the injection site (frequently during intravenous administration). The consequences of RPE depend on several factors, namely the physical characteristics of the radionuclide (e.g., energy, half-life, and type of emissions), properties of the radiopharmaceutical (e.g., pH, viscosity, osmolality, and adjuvants), volume of injection, fraction of the activity that was extravasated, site of RPE, and multiple patient-related factors (*1*,*2*). There is a potential risk of physical harm to the patient, especially with RPE of therapeutic radiopharmaceuticals (*2*). Further, insufficient delivery of the radiopharmaceutical to the target site may negatively impact image quality and its clinical interpretation, especially when quantitation is involved (*2**–**4*).

The detrimental effects of ionizing radiation on normal tissues have been described (*5*). The physical effects resulting from extravasation of chemotherapy drugs and intravenous iodinated contrast media are also well known (*1*,*6*). However, the unique situation with diagnostic radiopharmaceuticals is that they typically involve injections of lower volumes (∼0.5–1 mL, compared with >50 mL for iodinated contrast medium), with no direct cytotoxic effect of the pharmaceutical component (compared with the intrinsic cytotoxicity of chemotherapy) and much lower absorbed radiation doses (compared with external-beam radiation therapy) (*1*,*2*,*5*,*7*). A 2017 systematic review of RPE noted a major deficiency in the literature on the adverse clinical effects of RPE involving diagnostic radiopharmaceuticals, especially with regard to lack of clinical follow-up (*2*). The authors reported studies with a total of 3,016 cases of diagnostic RPE, out of which only 3 (0.1%) had any follow-up data available. It has been hypothesized that diagnostic RPE can lead to potential complications, either due to the volume effect (e.g., local hematoma and phlebitis) or due to the local effects of radiation (e.g., ulceration and desquamation) (*2*,*8*). However, in the absence of any systematically performed study in this space, the association of any adverse clinical effects with diagnostic RPE remains unknown.

Most cases with diagnostic RPE (85.7%) have been reported with ^99m^Tc-methylene diphosphonate (^99m^Tc-MDP) used for skeletal scintigraphy, probably because of the high volume of these studies and the acquisition of whole-body images that frequently capture the injection site (*2*). Therefore, we sought to review ^99m^Tc-MDP whole-body bone scintigraphy (WBBS) studies with RPE to determine the occurrence of any clinical adverse events in the patients. The primary objective of this study was to determine whether ^99m^Tc-MDP RPE in patients undergoing WBBS is associated with adverse clinical events, in the short term or the long term. Secondary objectives were to estimate the rate of RPE in the ^99m^Tc-MDP WBBS studies performed at the hospitals associated with our institute and to assess the requirement for a repeat scan due to insufficient diagnostic quality of the images.

## MATERIALS AND METHODS

**FIGURE 1.**
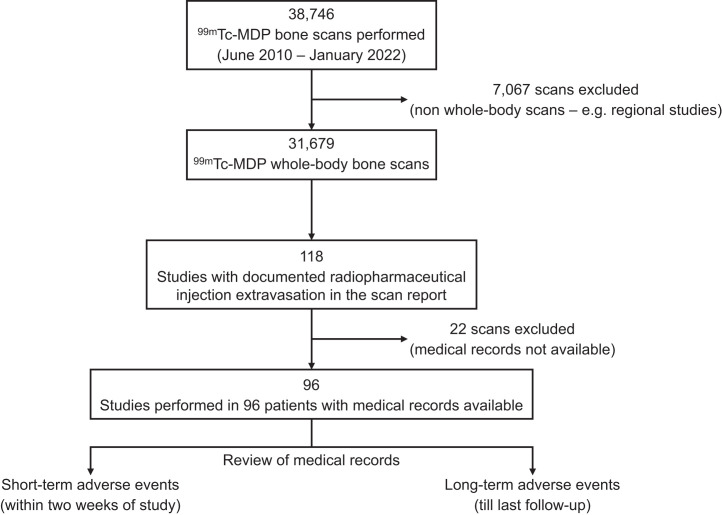
Study workflow.

We retrospectively reviewed the records of ^99m^Tc-MDP bone scans performed over 12 y (June 2010 to January 2022) at our medical center to identify WBBS studies for which the scan report documented RPE. The requirement for informed consent was waived for this retrospective analysis. Bone scans other than WBBS, such as limited-field-of-view regional studies, were excluded. The clinical records of patients with a documented RPE during ^99m^Tc-MDP WBBS were extensively reviewed for any related short-term adverse events (within 2 wk of the study), including local symptoms; any clinical documentation of the RPE (other than radiology and nuclear medicine); and care sought for dermatologic, neurologic, or musculoskeletal issues related to the site of RPE. If no results were found within the 2-wk duration, the next available clinical encounter closest to the scan date was reviewed. Medical admissions, if any, sought after the scan were also reviewed to determine the indication and whether it was related to the RPE. Medical records were also searched for long-term adverse events (through the date of last follow-up) to look for any local deleterious effects and related consults for dermatology, plastic surgery, oncology, or orthopedics. The study workflow is shown in Figure 1.

The Division of Nuclear Medicine at our institute currently routinely uses a small-gauge (23 or 25) butterfly needle (winged infusion set) for intravenous injections of ^99m^Tc-MDP. This policy was instituted in September 2017, before which a straight stick technique was more routinely used for intravenous injections. A previously placed peripheral intravenous line may also be used after confirming its patency. An easily palpable superficial vein in the antecubital fossa is the preferred site of cannulation. Proper placement of the needle in the lumen of the vein is verified by confirming adequate blood return before injecting the radiopharmaceutical. Under current institutional policy, if an RPE occurs, the incident is reported to the nuclear medicine physician for further guidance and documented in an online safety report. The nuclear medicine physician then assesses the severity of the RPE by its impact on the patient in terms of likelihood of physical harm and on the study in terms of image quality. It is then determined whether the patient requires further clinical evaluation and whether a repeat study is required.

## RESULTS

In total, 38,746 ^99m^Tc-MDP bone scans were performed over approximately 12 y (June 2010 to January 2022), of which 31,679 were WBBS. RPE was documented in 118 scan reports (0.37%). Medical records were available for 96 of these studies (performed on 96 patients), which formed the final cohort. The medical records of these 96 patients (mean age, 63.8 ± 14.3 y; 48 men, 48 women) were reviewed, with a median follow-up period of 18.9 mo (interquartile range, 7.8–45.7 mo). At the last follow-up, 76 patients were alive and 20 were deceased. The ^99m^Tc-MDP WBBS studies were performed for initial staging (*n* = 26), restaging (*n* = 18), or evaluation of osseous disease (*n* = 52). All scans except one were performed for an oncologic indication, the most common of which was prostate cancer (35.4%), followed by breast cancer (32.3%) (Table 1). The most common site for the radiopharmaceutical injection was the antecubital fossa (79.2%), followed by the hand or wrist (19.8%). The radiopharmaceutical was injected directly into the central venous catheter in 1 patient. The injections were performed on the left side in 50 patients and on the right side in 46 (including the injection in the central venous catheter). The mean injected activity of ^99m^Tc-MDP was 791.8 ± 59.2 MBq (21.4 ± 1.6 mCi).

**TABLE 1.** Primary Clinical Indication for ^99m^Tc-MDP WBBS in Which RPE Was Reported

Primary clinical condition for bone scan	*n*	%
Prostate carcinoma	34	35.4
Breast carcinoma	31	32.3
Hepatocellular carcinoma	10	10.4
Renal cell carcinoma	4	4.2
Lung carcinoma	3	3.1
Rectal carcinoma	2	2.1
Neuroendocrine carcinoma	2	2.1
Ovarian carcinoma	2	2.1
Colon carcinoma*	1	1.0
Urinary bladder carcinoma	1	1.0
Melanoma	1	1.0
Esophageal carcinoma	1	1.0
Pancreatic carcinoma*	1	1.0
Osteosarcoma	1	1.0
Rhabdomyosarcoma (cheek)	1	1.0
Paget disease	1	1.0

* One patient had both breast and pancreatic cancer, and one had both breast and colon cancer.

### Short-Term Adverse Events

**FIGURE 2.**
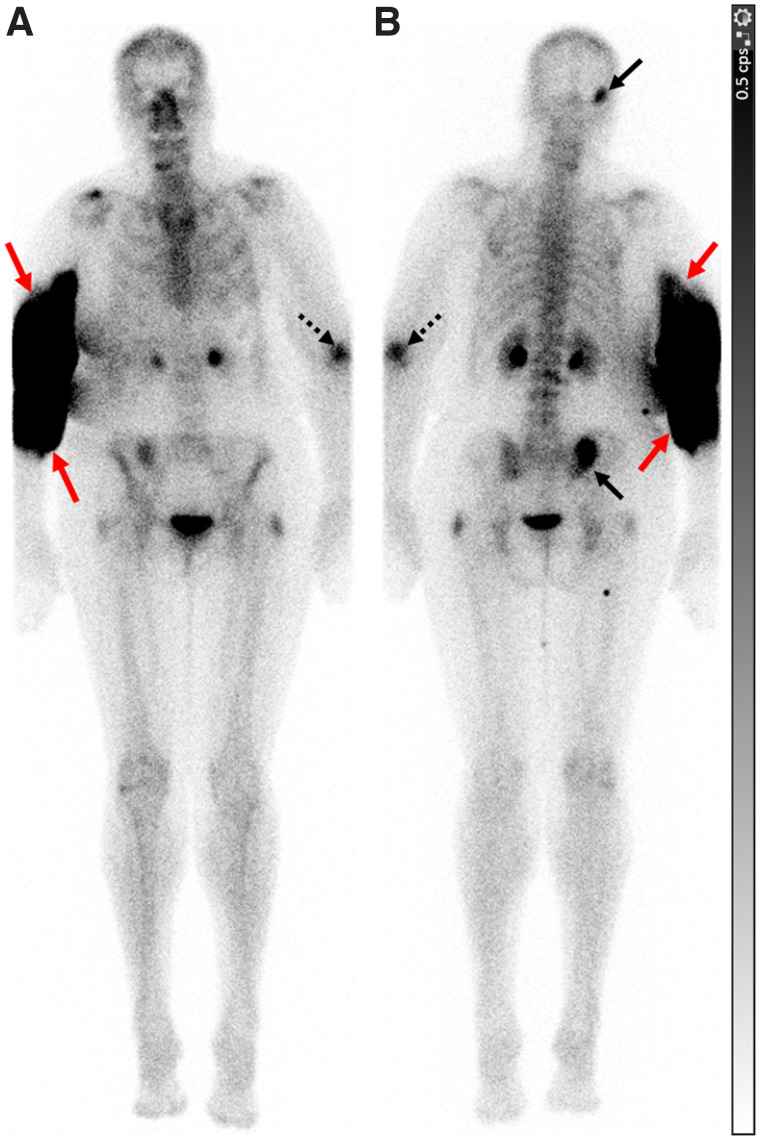
A 68-y-old woman with left breast cancer. WBBS was performed for restaging. Shortly after intravenous injection of 854.7 MBq (23.1 mCi) of ^99m^Tc-MDP in right antecubital fossa, patient complained of swelling and tenderness in her arm. Distal pulses and sensation in right upper extremity were intact. She had infiltration of iodinated contrast medium earlier in day in same arm while undergoing contrast-enhanced CT. She was recommended to use cold compresses and arm elevation at home and had experienced complete recovery when evaluated at next visit (4 wk). WBBS images in anterior (A) and posterior (B) projections show site of RPE around right elbow (red arrows). Patient had metastases to right occipital bone and right iliac bone adjacent to sacroiliac joint (solid black arrows). Prior fracture site in left elbow is also visualized (dashed black arrow).

There were 4 RPE-related events, of which 3 were symptomatic. Two patients experienced local discomfort with no blistering or erythema and intact distal pulses and sensation. One patient had local discomfort with tender swelling and intact distal pulses and sensation. One patient who remained asymptomatic was assessed to have normal grip strength and capillary refill. Of these 4 patients, 3 had an intravenous iodinated contrast infiltration previously on the same day while undergoing contrast-enhanced CT (Fig. 2). The RPE occurred at the same site. These 3 patients were recommended to apply cold compresses with arm elevation at home. The recommendation of cold compresses (over hot compresses) was made in view of the high-volume intravenous contrast extravasation earlier during the day of the scan—a volume that was much higher than that of the RPE. Two of these patients had a documented complete resolution of symptoms (one on the same day and the other within a week). An update on the clinical status was not documented for the third patient. The patient with RPE without any prior contrast extravasation did not require any active intervention, and his symptoms resolved on the same day. No appointments or referrals were made for any of these patients with primary care, dermatology, or plastic surgery. None of the patients had any severe RPE-related adverse events that required urgent care or hospital admission.

### Long-Term Adverse Events

No long-term local adverse events were observed in 88 of 96 (91.7%) patients. No appointments or referrals were made for any of these 96 patients (with dermatology, plastic surgery, oncology, or orthopedics) related to the RPE. Eight patients experienced adverse events, none of which could be directly attributed to the RPE (Table 2). The most common diagnosis in 3 of these 8 patients was carpal tunnel syndrome. Two patients had a temporary symptom arising from non–RPE-related factors (thrombophlebitis after an intravenous peripheral catheter placement, and contact dermatitis). One patient developed weakness of the upper extremity due to brain metastases from primary renal cell carcinoma. Two patients had paraesthesia in their arm, attributed to cervical radiculopathy in one patient and with an unknown etiology in the other.

**TABLE 2.** Long-Term Local Adverse Events (Irrespective of Etiology) in Patients with RPE Documented on Their ^99m^Tc-MDP WBBS Report

Injection site	Short-term symptoms*	Long-term symptoms on follow-up	Site	Scan to symptom onset (mo)	Diagnosis	Intervention	Resolution
R wrist	No	Pain, tenderness	Both hands (L > R)	6	Carpal tunnel syndrome, arthritis	Corticosteroids	No
L ACF	No	Paraesthesia	L arm	0.6	None	None	No
L ACF	No	Numbness	Both hands	33.6	Carpal tunnel syndrome	Surgery	Yes
L ACF	No	Paraesthesia	L arm	47.4	Cervical radiculopathy	None	No
L ACF	No	Weakness	L upper limb	0.6	Brain metastases	Corticosteroids	No
L ACF	No	Swelling, pain	L arm	0.7	Thrombophlebitis	Antibiotics	Yes
R ACF	No	Pain, weakness	R arm	2.8	Carpal tunnel syndrome	Corticosteroids	Yes
R hand	No	Rash, Itching	R arm	1	Eczema/contact allergy	Corticosteroids	Yes

* Within 2 wk of ^99m^Tc-MDP WBBS.

ACF = antecubital fossa.

**FIGURE 3.**
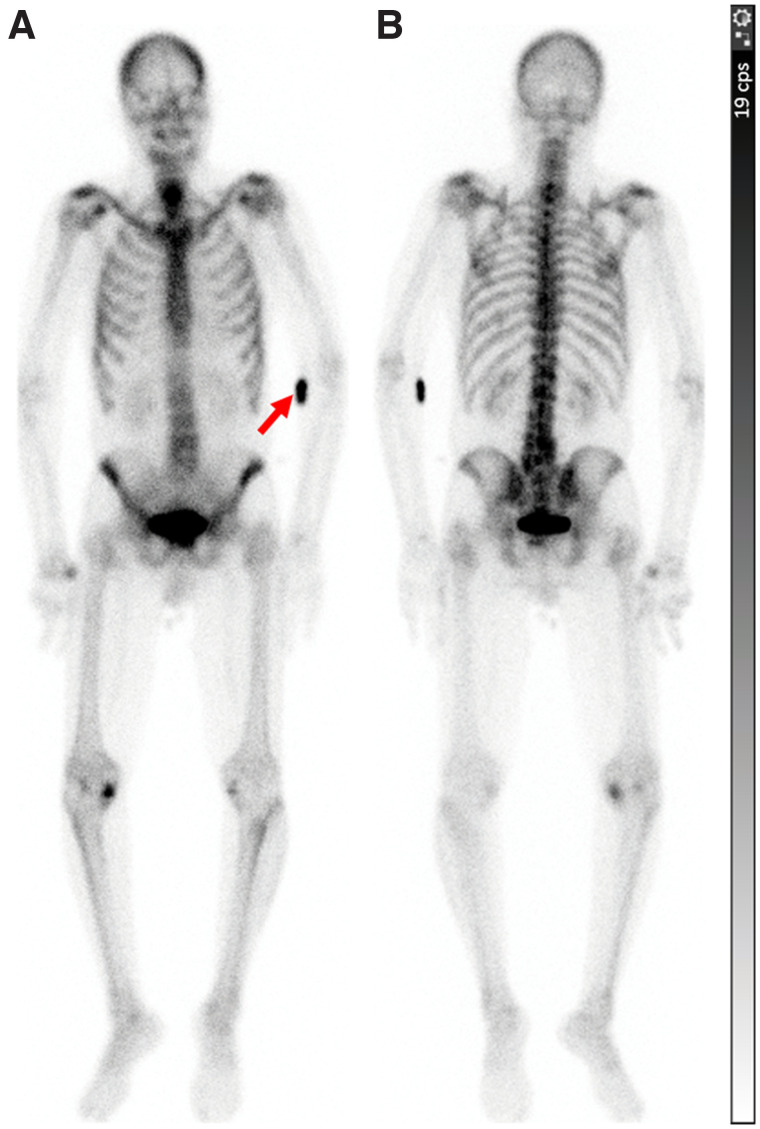
A 71-y-old man with prostate cancer. WBBS was performed for metastatic workup. Around 3 h after intravenous injection of 839.9 MBq (22.7 mCi) of ^99m^Tc-MDP in left antecubital fossa, images were acquired in anterior (A) and posterior (B) projections. RPE was noted at injection site around left elbow (arrow). Study showed increased radiopharmaceutical activity bilaterally in shoulder and knee regions, likely due to degenerative changes. No site suggestive of metastatic disease was seen. Scan was deemed to be of adequate diagnostic quality, and repeat study was not required.

Of the 96 studies with documented RPE, 93 (96.9%) were deemed adequate for clinical interpretation and rescanning was not recommended (Fig. 3). A repeat scan was recommended for 3 patients because of the suboptimal diagnostic quality of the initial scan with RPE. A repeat scan was subsequently performed uneventfully for 2 patients.

## DISCUSSION

Several prior studies have reported RPE with ^99m^Tc-MDP or other radiopharmaceuticals for skeletal scintigraphy; however, none described any clinical adverse effects associated with the RPE (*2*). Most prior discussions have focused on the extraskeletal distribution of the radiopharmaceutical after RPE (*9**–**13*). In the absence of any significant clinical follow-up data after RPE during diagnostic studies, it has been hypothesized that clinical adverse events might occur but are underreported (*2*). Our results provide evidence that clinical adverse events reported after ^99m^Tc-MDP RPE in patients undergoing WBBS are, in fact, rare. Among 96 patients with an RPE documented in the clinical report, only 3 had a short-term clinical adverse event. Of these, only one was potentially directly related to the radiopharmaceutical, whereas the others were almost certainly due to the volume or vesicant effects of an intravenous contrast extravasation earlier during the day.

Among the long-term local adverse events irrespective of etiology, the most common diagnosis was carpal tunnel syndrome (3/8 patients). Carpal tunnel syndrome has a complex pathophysiology, with several genetic and environmental factors involved (*14*). Its prevalence in the general population has been reported to be approximately 3%–5%, with higher rates in workers in specific industries (*15*,*16*). The rate of carpal tunnel syndrome in patients with reported RPE in our study was 3%, which conforms to the expected rate in the general population. With a median follow-up of over 18 mo (longest: >8 y), we did not find any long-term local adverse events that could be related to the RPE. This is a significant addition to the existing literature, as most prior studies did not report any follow-up of the patients with RPE. A systematic review published in 2017 showed that only 3 patients of 3,016 (0.1%) with a diagnostic RPE had any follow-up data reported (*2*). Two case reports included in the review describe ulcer development in 2 patients (after 2 and 3 y) after RPE of ^201^Tl-thallous chloride, although an attempt to establish causation to a radiation-induced injury was not made. One report described an erythematous pruritic plaque after RPE of ^131^I-iodocholesterol (*2*).

Prior studies have reported RPE rates ranging from 2% to 16% for PET/CT (*17*). Specifically, for ^99m^Tc-MDP, a study of 225 consecutive WBBS studies across 9 sites in Canada showed a 15% rate of RPE (*18*). The RPE rate did not change significantly after an educational intervention (postintervention RPE rate, 20%). Notably, they reported that the RPE did not limit clinical interpretation in any of the 450 studies. Another study of 2,435 ^99m^Tc-MDP WBBS scans performed between 1987 and 1994 reported ipsilateral axillary node visualization with RPE in 2% of the scans (*12*). In the present study, RPE was documented in the scan reports of 118 of 31,679 (0.37%) ^99m^Tc-MDP WBBS studies. A repeat study was recommended in 3 of the 96 patients with RPE documented in their reports. Our findings are similar to those of a Canadian study showing no impact of ^99m^Tc-MDP RPE on the clinical interpretability of 450 scans (*18*).

Mitigation of the effects of RPE is a relatively unexplored domain. Several techniques such as hot or cold compresses, injection of hyaluronate or steroids, and surgical interventions have been proposed to manage RPE (*2*). However, most have relied on the information gained from extravasation of chemotherapy drugs or iodinated contrast medium and extrapolated it to RPE. These extrapolations typically fail to account for the widely different mechanisms of tissue injury with cytotoxic agents and iodinated contrast media (*1*,*7*,*19*). Although some of these approaches may be useful for therapeutic radiopharmaceuticals, they are likely not required for diagnostic RPE (*2*). It has previously been recommended that RPE with a ^99m^Tc-labeled radiopharmaceutical does not require an active intervention (*8*). In the present study, 3 patients with RPE who had a prior iodinated contrast medium extravasation on the same day were recommended to use cold compresses and limb elevation at home. Cold compresses produce vasoconstriction, limit local inflammation and edema, and are effective to prevent contrast medium extravasation–related local injuries (*7*). The volume of RPE in these patients was minimal compared with the volume of extravasated contrast medium. Therefore, it was deemed appropriate to follow the guidelines related to contrast medium extravasation (*6*,*7*). Physiologically, the low bolus volumes of diagnostic RPE (∼0.5–1 mL) are unlikely to cause the volume-related adverse effects that are commonly seen with contrast media (∼50–200 mL) and chemotherapy infusions (*20*). The best technique to mitigate RPE-related adverse effects, however rare, is to prevent RPE. The major source of adverse events in our study was injection of the radiopharmaceutical at the same site as a prior intravenous contrast medium injection. It may be useful to ask whether the patient has had a recent intravenous contrast injection (or extravasation) and avoid using that site if injection at another site is feasible and appropriate.

Our study had certain limitations. We chose to identify RPE by searching the scan reports instead of visually reviewing the images of 31,679 WBBS studies. We recognize that this approach has the possibility of missing studies in which an RPE occurred but was not documented in the report. We did not perform any dosimetry estimations in the current study, as we did not have the time–activity curve data, nor were such estimations directly contributory to our primary objective. Prior studies have proposed methods for estimating local absorbed radiation dose as a surrogate for adverse clinical events (*21*). Since our approach was to directly look for any adverse clinical events in the patients with RPE, additional information, if any, provided by the dosimetry calculations would have been minimal. We assessed the short-term and long-term adverse events based on a comprehensive review of the medical records instead of interviewing each patient individually. Although our approach may have the potential to miss out on minor details, it is unlikely that a clinically significant adverse event would not have been documented in the medical records. It is also possible that a fraction of patients was not followed up at our center and that an adverse outcome could have been missed by chart review. As one of our secondary objectives, we chose the repeat-scan rates as a surrogate for diagnostic image quality. Although it would have been ideal to have a repeatability experiment with imaging performed “with RPE” and “without RPE” to assess the impact of RPE on image quality, it was outside the scope of the present study. Nonetheless, the repeat-scan rate is a useful real-world metric, as the interpreting physicians are likely to order a repeat scan if the image quality is suboptimal for clinical interpretation.

Despite these limitations, to our knowledge our study still included the largest cohort of ^99m^Tc-MDP WBBS studies performed over 12 y and reviewed for RPE-related adverse clinical events with a comprehensive follow-up. Our approach of assessing short-term and long-term adverse events ensured that any anticipated acute and chronic radiation-related injuries were accounted for. Future studies can include visual image review, comparing it with scan reports to determine the RPE rates. Quantitative techniques, including dosimetry, potentially might be used to assess the severity of RPE and correlate it with image quality and occurrence of any adverse events. However, given the rarity of adverse events, such efforts would seem to be of limited yield. The impact of specific interventions, including educational sessions and audits on the RPE rates, should also be explored, as well as the frequency of RPE with other nuclear medicine studies, though we expect that the results would be similar to our observations.

## CONCLUSION

Adverse clinical events, both acute and chronic, are exceedingly rare in patients with ^99m^Tc-MDP RPE during WBBS. Most cases of RPE were not associated with any clinical symptoms and did not require any active intervention. Those few RPE cases with symptoms appeared to be related to injection after intravenous contrast administration extravasations.

## DISCLOSURE

No potential conflict of interest relevant to this article was reported.

## Notes

KEY POINTS **QUESTION:** Is ^99m^Tc-MDP RPE in patients undergoing WBBS associated with adverse clinical events? **PERTINENT FINDINGS:** In this retrospective study of 31,679 ^99m^Tc-MDP WBBS performed over 12 y, we showed that RPE was documented in 118 patients. Of the 96 patients with available medical records, none had a long-term local adverse clinical event attributable to the RPE. Three patients had short-term adverse events, two of which likely resulted from prior intravenous contrast medium extravasation. **IMPLICATIONS FOR PATIENT CARE:** Adverse clinical events are rare in patients with ^99m^Tc-MDP RPE while undergoing WBBS. No long-term local clinical adverse event attributable to the RPE was observed.
